# Frequency and Severity of Temporomandibular Disorders Among Weightlifters at Fitness Centers in a Subset Population of Saudi Arabia: A Cross-Sectional Observational Study

**DOI:** 10.7759/cureus.49113

**Published:** 2023-11-20

**Authors:** Azzam Mansour Ibrahim Alrowdan, Syed F Mohsin, Shahzad Ali Shah, Tamim S Alkhalifah

**Affiliations:** 1 Dentistry, Qassim University, Ar Rass, SAU; 2 Oral Maxillofacial Surgery and Diagnostic Sciences, Qassim University, Ar Rass, SAU; 3 Conservative Dentistry and Endodontics, Qassim University, Ar Rass, SAU

**Keywords:** myofascial pain, temporomandibular joint, athletic, weight lifters, temporomandibular disorders

## Abstract

Background

Teeth clenching in weightlifters is a common finding that may result in temporomandibular disorders (TMDs). This study aimed to evaluate the severity and frequency of TMDs among weightlifters at fitness centers in Saudi Arabia.

Methods

A cross-sectional study was designed to collect data from weightlifters at fitness centers. A non-probability convenient sampling technique was applied using a valid and reliable Fonseca's questionnaire on a Google Sheet (Google, Mountain View, CA) to collect participant data. The survey was conducted from November 2022 to April 2023. Epi Info software (CDC, Atlanta, Georgia) was used to calculate the sample size, and a minimum sample of 278 was required. The data underwent analysis using SPSS version 20 (IBM Corp., Armonk, NY).

Results

Data analyzed from 375 participants revealed that 192 (51.2%) had mild signs of TMD, whereas 128 (34.13%) of the respondents had no symptoms of TMD. A significant difference was observed among female participants in all the temporomandibular joint severity categories. A statistically significant difference was observed between both genders concerning frequent headaches, earache, and nervousness.

Conclusion

The prevalence of TMD is high worldwide. Unsupervised athletic activity may result in the occurrence of TMD. Fonseca's questionnaire findings reveal a mild prevalence of TMD in weightlifters.

## Introduction

The temporomandibular joint (TMJ) is a true synovial joint with movements of a class III lever that consists of groups of muscles of mastication and cartilages that correlate synchronously to produce mandibular movements. The TMJ comprises the mandibular condyle, an internal fibro-cartilaginous disc that divides the fossa into two compartments, and the glenoid fossa of the skull's temporal bone [1,2]. Mandibular movement is primarily dependent upon the muscles of mastication [2,3]. Rapid opening of the mouth or opening against resistance also needs the assistance of the digastric, suprahyoid, infrahyoid, and mylohyoid muscles [4]. Mouth opening is achieved due to two distinct condyle movements in the glenoid fossa rotation and translation. Initial mouth movement of 15 to 20 mm occurs due to pure rotation that allows the mandibular condyle to rotate on a fibro-cartilaginous disc. After this stage, further opening is achieved by the contraction of the pterygoid muscles, leading the condyle and disc together to translate forward [4,5]. The temporomandibular ligaments provide the chief ligament restraint of movements, which resists the mandible's lateral motion [5].

Temporomandibular disorders (TMDs) are pain disorders of the masticatory system [6]. Pain, limited jaw movements, and TMJ clicking with or without reduction are the usual signs and symptoms of TMD. Clinical diagnoses of TMD are based on the established criteria of physical assessment, psychosocial status, and pain-related disability [7].

During a body workout, the mandible moves synchronously with the head movements, and it is challenging to consider the mandible in the rest or other relaxed positions. Therefore, It is optimal during exercise that the mandible be fixed in an optimal position [8]. During workouts, the condylar point can be displaced in the sagittal plane's backward and downward directions when strong force is exerted by the digastric and other back muscles that contract simultaneously and forcefully [9]. Studies have been done based on the association between masticatory forces and sports performance [8,10-13]. Muscle workouts have improved performance when weightlifting exercise is performed while the teeth are clenched together during mandibular fixation [8,14].

An electromyogram is a tool to detect mandible muscle movements during exercise. It has been demonstrated during a teeth clenching activity, but its accuracy was not precise due to its unstable sensors on human skin during training [14,15].

Sports and healthcare-related studies are increasingly focusing on several specific devices. Many studies have been conducted to determine the effectiveness of wearable sensors of the mouth guard type, in particular the acceleration sensor, designed for people suffering from concussions [16,17].

This cross-sectional study aimed to assess the severity and frequency of TMDs among weightlifters at fitness centers using Fonseca's questionnaire.

## Materials and methods

A cross-sectional observational study was conducted at various fitness centers to collect data from weightlifters. Informed consent was obtained from all the participants prior to the commencement of the study. A non-probability convenient sampling method was used to collect data from November 2022 through April 2023 using a valid and reliable Fonseca's questionnaire on a Google Sheet (Google, Mountain View, CA). To preserve the original English version of the translation, the authors translated the questionnaire into Arabic and adapted it to English.

To determine the sample size, Epi Info software version 5.4.1 (CDC, Atlanta, Georgia) was used; a minimum sample size of 278 was required, assuming a prevalence of 50% and a margin of error of 5% at a confidence level of 95%. Ethical approval for this study was granted by the Qassim University Ethical Review Board (ERB) (number: 22-10-05).

Inclusion and exclusion criteria

All male and female individuals between 15 and 65 years old, who practiced weightlifting and extensive training were eligible to participate in the study. To prevent bias, individuals with postural problems and no history of weightlifting were excluded.

Data collection method

Those participants who met the inclusion and exclusion criteria were invited to participate in the study. All participants were informed about the study's objectives and procedures, and their identities were kept confidential throughout the study. As far as the procedures are concerned, an interview was conducted to collect personal information, including age, time spent practicing the sport, the number of times they practiced it per week, and whether or not they were competitors.

The questionnaire consisted of 10 questions about TMD symptoms, and the participant is required to indicate whether the symptoms occur always, occasionally, or never. To score the questionnaire answered by the participant, 10 points were assigned to those individuals who answered "always" for every symptom, five points were given to those who responded "sometimes" for the investigated situation, and zero points were assigned to those who answered "never" for the symptom. To calculate the final score, they were added together. According to the final score on the questionnaire, there was no TMD where the score ranged from 0 to 15. However, between 20 and 40, there will be an indication of mild TMD. A moderate TMD was defined as 45 to 65, and a severe TMD was defined as 70 to 100.

Statistical analysis

Data were entered and analyzed using SSPS version 20.0 (IBM Corp., Armonk, NY). Demographic details such as age and gender and Fonseca's questionnaire were presented as frequencies and percentages, whereas weight and height were documented as means and standard deviations. The severity of TMDs using clinical index classification was also presented as frequencies and percentages. A chi-square test was applied to find out the association among weightlifters for Fonseca's questionnaire. Furthermore, a chi-square test and Pearson's correlation were used to determine the correlation between no TMD and TMD categories. A p-value of < 0.05 was considered statistically significant.

## Results

A total of 375 questionnaires were distributed among weightlifters. There were 240 (64.0%) male and 135 (36.0%) female respondents. The respondents were distributed into six age groups as follows: 52 (13.9%) in 15-20 years, 207 (55.2%) in 21-30 years, 69 (18.4%) in 31-40 years, 35 (9.3%) in 41-50 years, 10 (2.7%) in 51-60 years, and only two (0.5%) in above 60 years. The mean weight and height of the respondents were 74.01 ± 27.32 kg and 167.37 ± 11.62 cm, respectively, as shown in Table 1.

**Table 1:** Demographic details of weightlifters (n = 375).

Variable		Mean ± SD, n (%)
Age (years)	15-20	52 (13.9%)
	21-30	207 (55.2%)
	31-40	69 (18.4%)
	41-50	35 (9.3%)
	51-60	10 (2.7%)
	60 and above	2 (0.5%)
Gender	Male	240 (64.0%)
	Female	135 (36.0%)
Weight (kg)		74.01 ± 27.32
Height (cm)		167.37 ± 11.62

TMDs among weightlifters using Fonseca's questionnaire revealed that 66 (17.6%) respondents had frequent headaches, 171 (45.6%) respondents had not complained of frequent headaches, and 138 (36.8%) felt it sometimes. Most respondents (314, 83.7%) replied that they open their mouths without difficulty, and 55 (14.7%) respondents complained sometimes. Most of the respondents (325, 86.7%) can easily move their mandible from side to side. More than half of the respondents (244, 65.1%) replied that they did not get tired or have muscular pain while chewing, whereas 22 (5.9%) responded yes, and 109 (29.1%) replied that they sometimes get tired or have muscular pain while chewing. Nearly half of the respondents (147, 49.1%) had no pain on the nape, while 44 (11.7%) respondents had pain, and 184 (39.2%) had it sometimes. About 31 (8.3%) respondents had earaches or pain in the craniomandibular joints, whereas 273 (72.8%) had no pain and 71 (18.9%) had it sometimes. Only 42 (11.2%) respondents noticed TMJ clicking while chewing, whereas 262 (69.9%) respondents did not notice any TMJ clicking while chewing or when they opened their mouth, and 71 (18.9%) had it sometimes. Only 42 (11.2%) respondents clenched their teeth, 246 (65.6%) respondents did not clench, and 87 (23.2%) did sometimes. About 138 (44.0%) respondents replied that they did not articulate their teeth well, while 165 (36.8%) did articulate well, and 72 (19.2%) did sometimes. About 113 (30.1%) respondents considered themselves nervous people. On the other hand, 88 (45.6%) respondents did not, and 174 (36.8%) did sometimes, as shown in Table 2.

**Table 2:** The prevalence of temporomandibular disorders among weightlifters using Fonseca's questionnaire.

Fonseca's questionnaire			n	(%)
Do you have frequent headaches?				
		Yes	66	17.6
		No	171	45.6
		Sometimes	138	36.8
Is it hard for you to open your mouth?				
		Yes	6	1.6
		No	314	83.7
		Sometimes	55	14.7
Is it hard for you to move your mandible from side to side?				
		Yes	14	3.7
		No	325	86.7
		Sometimes	36	9.6
Do you get tired/muscular pain while chewing?				
		Yes	22	5.9
		No	244	65.1
		Sometimes	109	29.1
Do you have pain in the neck or stiff neck?				
		Yes	44	11.7
		No	147	49.1
		Sometimes	184	39.2
Do you have earaches or pain in craniomandibular joints?				
	Yes		31	8.3
	No		273	72.8
	Sometimes		71	18.9
Have you noticed any TMJ clicking while chewing or opening your mouth?				
	Yes		42	11.2
	No		262	69.9
	Sometimes		71	18.9
Do you clench or grind your teeth?				
	Yes		42	11.2
	No		246	65.6
	Sometimes		87	23.2
Do you feel your teeth do not articulate well?				
	Yes		138	44.0
	No		165	36.8
	Sometimes		72	19.2
Do you consider yourself a tense (nervous) person?				
	Yes		113	30.1
	No		88	45.6
	Sometimes		174	36.8

Association of gender with Fonseca's questionnaire among weightlifters revealed that there was a statistically significant difference between both genders concerning frequent headaches (p < 0.001), having earaches or pain in craniomandibular joints (p = 0.008), and nervousness (p = 0.002). Additionally, difficulty in opening the mouth, movement of the mandible from side to side, tiredness and muscular pain while chewing, having pain on the nape, TMJ clicking while chewing, clenching or grinding teeth, and improper articulation of teeth were not significantly influenced by the gender among weightlifters (p > 0.05), as shown in Table 3.

**Table 3:** The prevalence and association of temporomandibular disorders with respect to gender.

Variables			Gender		P-value
			Male	Female	
			n (%)	n (%)	
Do you have frequent headaches?					
		Yes	31 (12.9%)	35 (25.9%)	<0.001
		No	131 (54.6%)	40 (29.6%)	
		Sometimes	78 (32.5%)	60 (44.4%)	
Is it hard for you to open your mouth?					
		Yes	5 (2.2%)	1 (0.7%)	0.399
		No	203 (84.6%)	111 (82.2%)	
		Sometimes	32 (13.3%)	23 (17.0%)	
Is it hard for you to move your mandible from side to side?					
		Yes	9 (3.8%)	5 (3.7%)	0.930
		No	209 (87.1%)	116 (85.9%)	
		Sometimes	22 (9.2%)	14 (10.4%)	
Do you get tired/muscular pain while chewing?					
		Yes	15 (6.3%)	7 (5.2%)	0.859
		No	157 (65.4%)	87 (64.4%)	
		Sometimes	68 (28.3%)	41 (30.4%)	
Do you have pain in the neck or a stiff neck?					
		Yes	23 (9.6%)	21 (15.6%)	0.215
		No	122 (50.8%)	62 (45.9%)	
		Sometimes	95 (39.6%)	52 (38.5%)	
Do you have earaches or pain in craniomandibular joints?					
	Yes		14 (5.8%)	17 (12.6%)	0.008
	No		187 (77.9%)	86 (63.7%)	
	Sometimes		39 (16.3%)	32 (23.7%)	
Have you noticed any TMJ clicking while chewing or opening your mouth?					
	Yes		22 (9.2%)	20 (14.8%)	0.248
	No		172 (71.7%)	90 (66.7%)	
	Sometimes		46 (19.2%)	25 (18.5%)	
Do you clench or grind your teeth?					
	Yes		32 (13.3%)	10 (7.4%)	0.217
	No		154 (64.2%)	92 (68.1%)	
	Sometimes		54 (22.5%)	33 (24.4%)	
Do you feel your teeth do not articulate well?					
	Yes		87 (36.3%)	51 (37.8%)	0.871
	No		108 (45.0%)	57 (42.2%)	
	Sometimes		45 (18.8%)	27 (20.0%)	
Do you consider yourself a tense (nervous) person?					
	Yes		60 (25.0%)	53 (39.3%)	0.002
	No		68 (28.3%)	20 (14.8%)	
	Sometimes		112 (46.7%)	62 (45.9%)	

**Figure 1:**
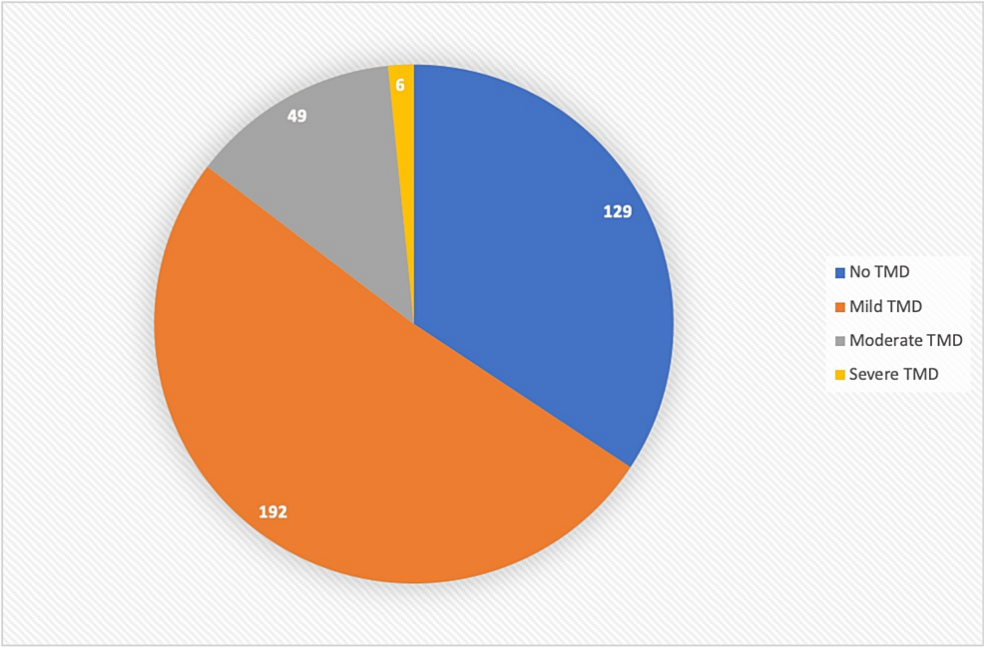
The severity of TMD using clinical index classification – Fonseca's questionnaire. TMD: temporomandibular disorder.

According to Fonseca's questionnaire score, 128 (34.13%) of the respondents did not have any signs of TMD, whereas 192 (51.2%) had symptoms of mild TMD, 49 (13.06%) with moderate TMD, and six (1.6%) with severe TMD dysfunction, as shown in Table 4 and Figure 1.

**Table 4:** The prevalence of severity of temporomandibular disorders (TMD) by using clinical index classification – Fonseca's questionnaire.

Variables	n (%)
No TMD (0-15 points)	128 (34.13%)
Mild TMD (20-40 points)	192 (51.2%)
Moderate TMD (45-65 points)	49 (13.06%)
Severe TMD (70-100 points)	6 (1.6%)

A comparison between males (n = 240) and females (n = 135) to the severity of TMDs revealed a statistically significant association among them (p = 0.027), as shown in Table 5.

**Table 5:** The association of temporomandibular disorders (TMD) severity with gender.

Variables	Male (n = 240), n (%)	Female (n = 135), n (%)	P-value
No TMD (0-15 points)	93 (38.75%)	35 (25.92%)	0.027
Mild TMD (20-40 points)	118 (49.1%)	74 (54.8%)	
Moderate TMD (45-65 points)	26 (10.8%)	23 (17.03%)	
Severe TMD (70-100 points)	3 (1.25%)	3 (2.2%)	

An association and correlation between no TMD and TMD categories indicated that a statistically significant weak positive correlation was observed between no TMD and with TMD (p = 0.021, r = 0.119*). Moreover, a statistically significant moderate positive correlation was observed between no TMD and mild TMD (p < 0.001, r = 0.486**). Additionally, a statistically insignificant weak positive correlation was observed between no TMD and moderate TMD (p = 0.162, r = 0.072), and a statistically negligible weak negative correlation was observed between no TMD and severe TMD (p = 0.136, r = -0.077), as shown in Table 6.

**Table 6:** The association and correlation between no TMD and TMD categories. TMD: temporomandibular disorder.

Variables	P-value	ρ
No TMD vs. with TMD	0.021	0.119^*^
No TMD vs. mild TMD	<0.001	0.486^**^
No TMD vs. moderate TMD	0.162	0.072
No TMD vs. severe TMD	0.136	-0.077

## Discussion

The contraction of the masticatory musculature during weightlifting exercises causes the subject to clench the mandible against the maxilla. This is usually performed by professional trainers and athletes, specifically during high-strength physical activity, resulting in myofascial pain, discomfort, and temporary withdrawal from the sports activity. TMDs are musculoskeletal disorders associated with pain, limited jaw movement, and TMJ sounds [18].

This study presented that 66 (17.6%) respondents had frequent headaches, 171 (45.6%) respondents had not complained of frequent headaches, and 138 (36.8%) felt it sometimes by using Fonseca's questionnaire. A study by Miettinen et al. revealed that 27.9% of trainers have facial pain [18]. Therefore, professional trainers need to be aware of this dysfunction and seek guidance for clinical examination and treatment options. The present study revealed that females have higher mandible movement than males; this finding is in line with Miettinen et al. who revealed that female athletes have high mandible movement, about 18.4%, compared to male athletes (13.4%) [18]. Increased frequency of mandible movement from side to side leads to fatigue of jaw muscles, leading to TMJ pain. Regarding the severity of TMD using the clinical index classification, this study showed that the prevalence of severity in the mild TMD group was 51.2%. In comparison, the lowest severity of TMD was 1.6%. A study by Kaminiecki and Davatz based on Fonseca's questionnaire in CrossFit revealed that among 52 subjects, 40.4% have symptoms, with 38.4% showing mild and 2.0% showing moderate TMDs [19]. Another study by Habib et al. showed that 53.2% of participants had no symptoms, while severe dysfunction was recorded in 1.1% [20].

The variation in the prevalence of TMDs can be attributed to gender, age, and sample size-related variations. In this study, a comparison between males and females in the severity of TMDs revealed a statistically significant association in female gender (p = 0.027). Several studies have investigated the gender differences in TMD prevalence and severity. In a survey conducted by Cabral et al., it was observed that signs and symptoms related to TMD were more prevalent among females than males [21]. A study from Saudi Arabia using Fonesca’s index and Zung anxiety scale found that 62.8% of female participants had high levels of anxiety in relation to TMD [22]. Another study from Saudi Arabia claims that females are more likely to experience TMD as compared to male participants [23]. The available evidence suggests a highly significant association between gender and the severity of TMD. Females tend to have a higher prevalence and severity of TMD than males. However, further research is needed to understand the underlying mechanisms and factors contributing to these gender differences in TMD severity.

Our study found a statistically significant moderate positive correlation between no TMD and mild TMD. According to a survey conducted by Kaminiecki and Davatz, it was observed that the intensity of TMD symptoms had a positive correlation with the duration of exercise. The study further indicated that mild TMD symptoms were reported among the 19 participants who engaged in physical activity for four months to one and a half years. Among the cohort of 22 individuals who engaged in a practice duration ranging from 1.5 to 2.5 years, it was observed that 41% had symptoms associated with TMD. Among the group consisting of 11 individuals who underwent training for a duration ranging from 2.5 to eight years, it was shown that 36% exhibited mild symptoms of TMD [19]. Another study revealed that TMD symptoms were less frequent in competitive female athletes (52.2%) than in recreational female athletes (63.0%); the reason could be qualified trainers properly supervise them [24].

Limitations

It is important to note that the sample size of this study was small. With regard to the interpretation of condylar resorption, particular investigations are required by using cone-beam CT and MRI. Further, in vitro studies should be planned using special devices that determine the actual values of occlusal forces.

## Conclusions

The overall frequency of TMD was 65%. Female gender and psychological status are associated with TMD. Based on Fonseca's questionnaire, mild TMD prevalence appears among weightlifters. Sports activities should be supervised by certified trainers and physiotherapists to reduce the incidence of TMD.

## Appendices

Questionnaire

Title: Frequency and severity of temporomandibular disorders among weightlifters at fitness centers in a subset population of Saudi Arabia. An observational cross-sectional study.

Gender:

Male

Female

Age:

<15

15-20 

21-30

31-40

41-50

50-60

60 above

What is your weight?

What is your height?

Questions: (No, Sometimes, Yes)

1. Is it hard for you to open your mouth?

2. Is it hard for you to move your mandible from side to side? 

3. Do you get tired/muscular pain while chewing?

4. Do you have frequent headaches?

5. Do you have pain in the neck or stiff neck?

6. Do you have earaches or pain in craniomandibular joints?

7. Have you noticed any TMJ clicking while chewing or when you open your mouth?

8. Do you clench or grind your teeth?

9. Do you feel your teeth do not articulate well?

10. Do you consider yourself a tense (nervous) person?
